# Local and Systemic Hemostatic Agents: A Comprehensive Review

**DOI:** 10.7759/cureus.72312

**Published:** 2024-10-24

**Authors:** Bardia Jamali, Saeed Nouri, Salimeh Amidi

**Affiliations:** 1 Research Center for Health Management in Mass Gathering, Red Crescent Society of the Islamic Republic of Iran, Tehran, IRN; 2 Research Center for Emergency and Disaster Resilience, Red Crescent Society of Islamic Republic of Iran, Tehran, IRN; 3 Department of Medicinal Chemistry, School of Pharmacy, Shahid Beheshti University of Medical Sciences, Tehran, IRN

**Keywords:** hemorrhage control, hemostatic agent, local hemostatic agent, surgery, trauma

## Abstract

Traumatic hemorrhage is the leading preventable cause of death worldwide. Systemic administration of hemostatic agents requires trained personnel and preparation, limiting their use in combat environments and prehospital settings. However, local administration of hemostatic agents may ameliorate these challenges. Currently available hemostatic products are limited by risk of infection, immunogenicity, tissue damage, limited usage and efficacy, high costs, short shelf life, and storage requirements under specific conditions. Future studies should be considered to overcome these limitations and develop effective, multifunctional hemostatic materials for widespread usage. In this review, we will provide an overview of the most commonly used systemic and local hemostatic agents in hemorrhage control.

## Introduction and background

Blood provides nutrients and removes waste products from the organs of the body. Hemorrhage is defined as the loss of blood for any reason from the damaged blood vessels, including arteries, veins, and capillaries [1-3]. Hemorrhage can be classified as internal or external, with three main causes: trauma, disease (e.g., liver disease, cancer, diabetes, vitamin K deficiency, peptic ulcer, hemophilia, and von Willebrand disease), and medications (e.g., aspirin, antiplatelets, and anticoagulants).

Hemorrhage severity is divided into classes I to IV according to the percentage of blood volume lost [1,4,5]. In class I hemorrhage, less than 15% of the total blood volume is lost, and the vital signs of patients are within normal limits. In class II hemorrhage, 15% to 30% of the total blood volume is lost, leading to a slight deviation from normal vital signs, including tachycardia, increased respiratory rate, as well as patient complaints of nausea, fatigue, and cooling of the extremities. In class III hemorrhage, 30%-40% of the total blood volume is lost, and further deviations from normal vital signs are observed. In class IV hemorrhage, more than 40% of the total blood volume is lost, resulting in tachycardia, hypotension, absent peripheral pulses, tachypnea, and a change in mental status.

Uncontrolled hemorrhaging leads to the production of lactic acid and, ultimately, metabolic acidosis, coagulopathy, organ failure, coma, and death. Coagulopathy, hypothermia, and acidosis are significant risk factors for hemorrhagic death [6-9]. The first critical step of prehospital care is the prevention and treatment of the lethal triad of hypothermia, acidosis, and coagulopathy besides hypocalcemia [10].

Epidemiology

According to the WHO, traumatic injury is one of the leading causes of death worldwide. It was reported that in 2016, over five million deaths due to trauma occurred annually, with this number expected to exceed 8 million by the year 2020 [8,10-13]. Mortality rates are higher in middle- and low-income countries, mainly due to traffic road accidents [10,12]. Hemorrhage secondary to traumatic injury is the principal preventable cause of death (approximately 35% of the global mortality rate) and disability in those injured [8-10,14-16]. As 50% of these deaths occur due to uncontrolled hemorrhage before reaching hospital, the control of bleeding is time-sensitive and can be challenging [10,14,15].

Treatment and management

Since ancient times, humans have attempted to control hemorrhage by spreading different kinds of topical agents over the affected area. A mixture of grease, barley, and wax was used as a hemostatic agent in ancient Egypt. Similarly, the ancient Greeks used hemostatic herbs to stop bleeding [17]. In the last two decades, despite advances in medicine and prehospital care, the mortality rate of traumatic hemorrhage has not improved significantly. Control of hemorrhage requires immediate intervention, which is provided through the application of direct pressure, packing open wounds with clean cloths or gauze, hemostatic dressings, and/or application of tourniquets. If hemorrhage persists and metabolic recovery is not possible, the lethal triad of hypothermia, acidosis, and coagulopathy will result in death [10,16].

In this review, we will provide an overview of the most commonly used systemic and local hemostatic agents in hemorrhage control, discuss their mechanism of action, compare their positive and negative aspects, and give recommendations for their use.

Methods

A broad literature search was conducted from April 20, 2024 to May 20, 2024 on PubMed and Scopus, without restrictions on the publication date. The search focused on human studies with available full-text, English-language articles. Keywords used in the search included 'topical hemostatic agents or dressing,' 'traumatic hemorrhage control,' 'hemostatic agents,' 'chemical hemostatic agents,' 'systemic hemostatic agents,' and 'topical hemorrhage control.' Many studies were found; titles and abstracts were read to determine whether the articles would be included in the review. Additional articles were chosen from the references listed in the original papers.

## Review

Systemic hemostatic agents

Fresh Frozen Plasma (FFP)

FFP contains fibrinogen, protein C, antithrombin, protein S, albumin, and tissue factor pathway inhibitors, and is used as a hemostatic agent to replenish coagulation factors lost during bleeding [18]. It can be used alone or in combination with RBCs.

It can be administered for the treatment of coagulation factor deficiencies with abnormal coagulation tests in active bleeding, warfarin-induced bleeding, and trauma patients requiring massive transfusion [19].

Some reported adverse effects of FFP administration are allergic and anaphylactic reactions, transmission of infectious agents, metabolic complications like citrate toxicity, and transfusion-related acute lung injury [19].

There is controversy about the efficacy of FFP. Most studies concluded that reduced mortality was associated with higher FFP ratios. However, Pragmatic Randomized Optimal Platelet and Plasma Ratios (PROPPR) randomized controlled clinical trials demonstrated no significant differences in mortality rate between FFP:platelet ratios of 1:1:1 and 1:1:2 [20-22]. The main limitations of this product are requirements for freezers and thawing equipment. As such, FFP is not ideal for use in battlefield or prehospital settings [18].

Prothrombin Complex Concentrate (PCC)

PCC is a plasma-derived factor concentrate that is available in two forms: four-factor PCC and three-factor PCC. The four-factor PCC contains factors II, VII, IX, X, and anticoagulant proteins C and S. The three-factor PCC is similar to four-factor PCC but without factor VII.

Retrospective and prospective studies have shown that a faster treatment of trauma-induced coagulopathy with PCC has no significant effects on mortality, indicating that more comprehensive clinical trials are required to prove the efficacy of PCC as a first-line therapy for patients with hemorrhage.

The main advantages of PCC are its ease of access in either prehospital care or at emergency admission, and the high concentration of factors in a small volume [18].

Dried Plasma (Lyophilized Plasma)

Dried or lyophilized plasma is commonly manufactured by lyophilization and less commonly by spray drying. French lyophilized plasma (FLYP), German lyophilized plasma (LyoPlas), and bioplasma freeze-dried plasma (FDP) are three commercially available dried plasmas [18,23]. FLYP is produced from approximately ten carefully screened and monitored donors by the French Blood Bank, where pathogen reduction is done using an amotosalen and ultraviolet light process. LyoPlas is manufactured from a single donor, who is quarantined for at least four months by the German Red Cross. The pathogen reduction is done by the solvent-detergent method.

Bioplasma FDP is manufactured from hundreds (up to 1500) donors by the National Bioproducts Institute of South Africa. The pathogen reduction is done by the solvent-detergent method. A retrospective cohort study demonstrated that the application of dried plasma in the prehospital setting has a positive effect on the coagulation profile [24]. The main advantage of lyophilized plasma is a lack of freezer or refrigerator requirements. However, lyophilized plasma is limited by regulatory issues, production logistics, and product issues [18].

Fibrinogen Concentrate (FC)

FC is a source of fibrinogen derived from 30-60,000 human plasma donors and is used to restore fibrinogen levels during bleeding to their normal physiological range of 2-4 g/L. Fibrinogen is a critical protein in the coagulation cascade and is a precursor of fibrin. It also binds to glycoprotein GPIIb/IIIa receptors on the surfaces of platelets and activates platelet aggregation. Below-normal fibrinogen levels are correlated with increased hemorrhage, coagulopathy, and poor clinical outcomes.

FC is currently approved for the treatment of bleeding in patients with congenital fibrinogen deficiency or uncontrolled bleeding in trauma patients.

Some reported adverse effects of FC administration are thromboembolic complications, allergic and anaphylactic reactions.

Commercially available FC products include Haemocomplettan or RiaSTAP (CSL Behring, Marburg, Germany), Fibrinogen HT (Benesis, Osaka, Japan), Clottafact (LFB, Les Ulis, France), Fibryga (Octapharma, Lachen, Switzerland), and FibroRAAS (Shanghai RAAS, Shanghai, China). According to placebo-controlled, randomized, prospective, double-blinded trials, prehospital administration of FC (Clottafact, LFB France) can restore early fibrinogen depletion, boost rapid blood clot initiation, and increase clot stability in hemorrhagic patients [18,23,25,26].

Cryoprecipitate

Cryoprecipitate is a traditional source of fibrinogen and is prepared from FFP. In contrast to FC, cryoprecipitate contains many other hemostatic coagulation factors in addition to fibrinogen; similar to FC, it is used to ameliorate hypofibrinogenemia during major hemorrhage. FC is preferred by physicians due to product standardization (vials contain 20 g/L fibrinogen), its ease of carry, viral inactivation, lack of required storage or thawing in a blood bank, and long shelf life [18,23,25].

Tranexamic Acid (TXA)

TXA is a synthetic antifibrinolytic agent that works by blocking the breakdown of blood clots. TXA competitively inhibits the activation of plasminogen to plasmin and noncompetitively inhibits plasmin from breaking down fibrin clots at higher concentrations. TXA may have beneficial effects independent of regulating fibrinolysis in injured patients. Clinical trials by WOMEN [27] and CRASH-2 [28] reported that TXA administration within three hours after injury reduced mortality in hemorrhagic patients. Recently, TXA has attracted more attention for prehospital use [18].

Platelets

Platelets are one of the components of blood with an essential role in clotting. To date, the role and efficacy of platelet transfusion during traumatic bleeding remain unclear due to practical issues. The specific storage conditions (i.e., room temperature and continuous agitation) and short shelf life (between 5-7 days) of platelets are two main challenges in the production of standard platelet concentrates (PCs) [23]. To overcome these challenges, different types of platelets and different techniques have been proposed. Cryopreservation (CRYO-PLTs) and cold storage are two techniques that have been proposed to prolong PC shelf life by decreasing bacterial proliferation and platelet metabolism. Cryopreserved platelets (CRYO-PLTs) are prepared by suspending platelets in dimethyl sulfoxide, freezing at -80 °C, and then thawing and resuspending by either plasma or saline. In France and a few other European countries, CRYO-PLTs are approved for widespread usage [23].

Cold-stored PCs (2-6 °C) are an alternative to CRYO-PLTs with simpler preparation and storage procedures. Cold-stored PCs have decreased bacterial contamination risks and improved efficacy compared with normal PCs. In 2015, the US FDA-approved cold-stored PCs for hemorrhage control in bleeding patients [23].

Lyophilization of human platelets is another technique to increase the ease of handling and shelf life of PCs. Human lyophilized platelets (dried platelets), which are prepared for intravenous infusion by the addition of sterile water, are easy to use and effective for the treatment of noncompressible hemorrhage [18,23]. To address the practical issues of platelet application for hemorrhage control, such as limited efficacy and portability, short shelf-life, antigen matching, and high risk of bacterial contamination, synthetic platelets were introduced as a novel solution. Synthetic platelets comprise liposomes and particles whose surfaces are modified with platelet-binding motifs or platelet surface adhesion peptides to mimic the process of platelet activation. These synthetic platelets have demonstrated enhanced hemostasis in animal model bleeding studies [18].

Synthetic Polymers

Intravenous synthetic polymers and nanoparticles with specific motifs for the activation of the coagulation cascade are of interest as hemostatic agents, as they have suitable half-lives and low costs. PolySTAT is a synthetic polymer comprising a linear poly (hydroxyethyl methacrylate) backbone grafted with fibrin-binding peptides. Studies conducted in vivo and in vitro have shown the efficacy of synthetic polymers in clotting blood, and potential application in transfusion to treat coagulopathy and reduce bleeding in animal models [18].

Local hemostatic agents

Systemic administration of hemostatic agents requires a qualified professional and adequate preparation time (i.e., rewarming refrigerated liquid products or reconstituting solid hemostats), which limits their use in combat environments and prehospital settings. However, local administration of hemostatic agents may ameliorate these challenges. Most local hemostatic agents are produced in the form of gauze or membrane sheets. They are also available in various forms including solutions, sponges, powders, foams, and gels for bleeding control (Table 1).

**Table 1:** Local hemostatic agents and their mechanism of action. References: [17,18,23,29,30]

Category	Hemostatic agent	Mechanism of action	Brand
Silicate hemostatic materials	Smectite	Procoagulant, Water absorption	Wound Stat
	Kaolin	Procoagulant, Water absorption	Quickclot Combat Gauze
	Zeolite	Procoagulant, Water absorption	Quickclot
Polysaccharide hemostatic materials	Chitosan	Procoagulant	HemCon, Celox, Celox Rapid
	Chitosan + Silica	Procoagulant	TraumaStat
	Chitosan + Cellulose	Procoagulant, Physical Barrier	Xstat
	Polysaccharide spheres (Starch)	Procoagulant, Water absorption	Arista AH, PerClot
	Oxidized regenerated cellulose (ORC)	Procoagulant, Water absorption	Traumastem, Surgicel Fibrillar, Surgicel Nu-Knit
	Alginate	Mucoadhesive agent	Kaltostat, Sorbalgon
Biologically derived homeostatic materials	Thrombin (Recombinant human)	Procoagulant	Recothrom
	Thrombin (Bovine)	Procoagulant	Thrombin-JMI
	Thrombin (Human-pooled plasma)	Procoagulant	Evithrom
	Fibrinogen + Thrombin (Fibrin Sealant)	Procoagulant	TachoSil, Tacho-Comb, Fibriseal, Tisseel, Crosseal, Quixil, Evarrest, Evicel
	Fibrinogen + Thrombin + Chondroitin Sulfate	Procoagulant	Hemoblast
	Collagen (Bovine)	Procoagulant	Ultrafoam, Avitene
	Microfibrillar Collagen	Procoagulant	Lyostypt, Avitene
	Microfibrillar Collagen + Thrombin (Bovine)	Procoagulant	Costasis
	Gelatin (Porcine)	Procoagulant	Gelfoam, Surgifoam, Spongostan, Stypro
	Gelatin + Thrombin (Human-pooled plasma)	Procoagulant	Floseal, Surgiflo
	Albumin (Bovine) + Glutaraldehyde	Mucoadhesive agent	Bioglue, PreveLeak
Synthetically derived hemostatic materials	Polyethylene glycol	Mucoadhesive agent	CoSeal, DuraSeal
	Cyanoacrylate	Mucoadhesive agent	Dermabond, Histoacryl, Glubran
Chemical hemostatic materials	Ferric Sulfate	Physical Barrier	ViscoStat
	Aluminum Chloride	Physical Barrier	ViscoStat clear

Silicate-based Materials

Zeolite, bentonite, kaolin, porous silica, and smectite are examples of silicate-based materials used for bleeding control as granules or impregnated gauze. The granule form is effective in low-pressure bleeding, whereas impregnated gauze is effective in high-pressure bleeding. Impregnated gauze can be applied at the wound site under pressure without being washed away by blood flow, as in granules.

Smectite is provided as granules with high-efficacy hemostatic properties, but the granules can enter the circulation and cause thrombosis. Therefore, this product is not recommended by the FDA for hemorrhage control. Kaolin, which began replacing zeolite in hemostatic products in 2008, is used for its absorbent properties and ability to activate factor XII in the coagulation cascade, accelerating clot formation. QuickClot® (Z-Medica, Wallingford, CT) is sold as a kaolin-impregnated gauze, which can be used in the prehospital setting and operating room for a wide range of wounds on the head, face, upper and lower extremities, and junctional regions [6,17,18,29-33].

Chitin and Chitosan

Chitosan is a biodegradable, viscoelastic, inert, biocompatible, natural, linear polysaccharide copolymer with D-glucosamine and N-acetyl-D-glucosamine monomers. The amino group in the polymer structure is protonated in the physiological pH range and can electrostatically interact with negatively charged cell membranes. Chitosan is obtained by the deacetylation of chitin, which is found in the cell walls of crustaceans, arthropods, shrimp, crabs, shellfish, some fungi, and algae. In addition to its hemostatic activity reported in the early 1980s, chitosan also has antibacterial and wound-healing activities. To date, numerous chitosan-based hemostatic products have been developed, including Celox® RAPID, Celox® Gauze, and ChitoGauze®, which are available in solutions, rolls, gauzes, and granule forms. The hemostatic efficacy and safety of chitosan-based hemostatic products have been demonstrated in many randomized controlled clinical trials. The efficacy of these products is not influenced by coagulopathy due to the direct electrostatic interaction of chitosan with the negatively charged cell membranes of erythrocytes.

Various modifications of chitosan’s structure have been suggested to improve its hemostatic property in hemostatic dressings. For example, quaternary ammonium groups can be added to chitosan’s structure, increasing the number of positively charged centers as well as blood cell adhesion and antimicrobial activity. The presence of quaternary ammonium groups in the chitosan structure can make the derivative toxic to normal cells.

Thiol-modified chitosan and catechol-modified chitosan have been synthesized to promote clotting. Specifically, the introduction of the catechol group to the structure of chitosan can cause vasoconstriction and prevent massive bleeding. Furthermore, introducing dodecyl groups to the chitosan polymer increases its hydrophobicity, which can increase the accumulation of RBCs and antibacterial activity. One potential limitation of chitosan-based hemostatic agents is the biodegradation of chitosan, which should be considered [18,33-37].

Expandable Polymer

Non-absorbable/absorbable, expandable polymer can quickly absorb blood and apply pressure by rapid expansion inside the wound cavity to stop bleeding. Expandable polymer hemostats are usually made with a cellulose polymer, either alone or combined with other hemostatic agents, and are available in sponge or powder forms [18,38]. XStat (Revmedx, Wilsonville, OR, USA) is available as a syringe filled with rapidly expanding cellulose sponges coated with chitosan. Clinical studies have shown that XStat is safe and effective for control of hemorrhage in penetrating trauma. The main limitation of non-biodegradable sponges is the need for removal after hemostasis. The presence of a tiny radiopaque marker in each sponge allows for their detection in the body via X-ray imaging [18,39].

Oxidized Regenerated Cellulose (ORC)

First introduced in 1960, ORC is a biodegradable agent derived from plant cellulose. ORC has acidic properties that can cause protein denaturation and exhibits both hemostatic and bactericidal effects. This agent is useful for capillary, venous, and minor arterial bleeding, and demonstrates a greater hemostatic effect when applied dry. ORC is available as Surgicel Fibrillar and Surgicel Nu-Knit by Ethicon, and Johnson and Johnson, respectively. Although the antimicrobial effect of ORC resulting from its low pH is one of its main benefits, this low pH limits the combination of ORC with other topical hemostatic agents (e.g., thrombin), increases inflammation in surrounding tissue, and delays wound healing [17,30,33,36,40].

Microporous Polysaccharide Hemospheres

Microporous polysaccharide hemospheres (MPH) are bio-inert, absorbable particles derived from purified plant starches, such as potato starch. When these products encounter blood, they absorb fluid and concentrate blood components. The aggregation of blood components forms a mechanical barrier to bleeding and activates the coagulation cascade. These products are effective within minutes and absorbed by the body within 24-48 hours. As these agents swell after fluid absorption, they are not recommended in some surgeries [41]. Marketed products include Arista AH, which was approved by the FDA for use in orthopedic surgery [30,42], and PerClot, which exhibits wound-healing through increased release of TGF-β1 [43].

Alginate

Alginate, obtained from seaweed, is a natural biocompatible polymer with negatively charged ions and low toxicity. Alginate can form a gel or crosslink to absorb fluids, with very good hemostatic and wound-healing properties, as the release of calcium ions from the crosslinked alginate can activate the coagulation process. Alginate acts as an occlusive dressing that creates a protective barrier for the wound. Alginate dressings work best on mild hemorrhage and are ineffective in high-pressure hemorrhage. These dressings can activate macrophages to accelerate wound healing [30,36]. Sorbalgon is one of the marketed products based on alginate [44,45].

Mineral Caustic and Astringent Agents

Caustic hemostatic agents such as aluminum chloride, ferric sulfate, Monsel’s solution, alum, silver nitrate, and zinc chloride paste act by coagulating proteins and enhancing thrombus formation. Some of these compounds have an astringent effect, leading to superficial and local coagulation. These agents are effective tools for hemorrhage control and can be applied to a wound using either gauze or a Q-tip applicator. Some of these agents can pigment the skin or form eschar.

Alum, or hydrated sulfate salts of aluminum and alkali metals, such as aluminum potassium sulfate (KAl(SO4)2), are slightly less effective hemostatic agents compared to epinephrine at 100% concentration, which has an astringent effect. Alum has been recommended as a substitute for epinephrine in controlling bleeding in gingival tissues, as it is safer and has fewer systemic effects [46]. Similarly, filter alum, or aluminum sulfate (Al2(SO4)3), is effective in controlling hemorrhage and is biologically acceptable [46].

Aluminum chloride (AlCl3) is one of the most commonly used caustic hemostatic agents with astringent properties. AlCl3 is used at concentrations of 5%-25%, with minimal systemic side-effects, and does not leave pigment on the skin or tissues. Popular AlCl3 products such as ViscoStat™ Clear are sold at concentrations of 20%-25% [46,47].

Trichloroacetic acid has a hemostatic effect and is used at a concentration of 35% as a liquid or gel on the gingiva [48].

Ferric subsulfate (Fe4(OH)2(SO4)5), or Monsel’s solution, is used as a local hemostatic agent in the form of solution or paste. Monsel’s solution was first introduced by Leon Monsel, a “pharmacien major” of the French army, in 1856. The US Pharmacopeia published a monograph for this solution in 1863. Monsel’s solution is a brown fluid that is prepared by reacting ferric sulfate with sulfuric acid and nitric acid. As the pH of this solution is approximately one, it can denature and agglutinate proteins such as fibrinogen; it is also corrosive, damages soft tissues and enamel, and stains the teeth and skin [46,49,50].

Ferric sulfate (Fe2(SO4)3) is one of the most commonly used caustic hemostatic agents with low astringent properties. Popular Fe2(SO4)3 products such as ViscoStat™ are available at concentrations of 15%-20%. These products do not damage tissue, and healing is more rapid than with AlCl3. However, concentrations above 15% can cause skin irritation, and pigment the tissue a yellow-brown or black for one or two days [46,51].

Calcium sulfate (CaSO4), as a hemostatic agent, is inexpensive, biocompatible, and resorbable with almost no inflammatory response [52,53].

Zinc chloride (ZnCl2 (bitartrate)), used at concentrations of 8% and 40%, has hemostatic and astringent effects. As this compound is escharotic and results in permanent tissue injury, its use is uncommon at present [46].

Tannic acid, used at concentrations of 20% and 100%, has astringent properties and minimal hemostatic effects [46].

Negatol solution, a 45% condensation product of metacresol sulfonic acid and formaldehyde, is highly acidic and used at concentrations of 10% and 100%.

Silver nitrate is an inorganic chemical with antimicrobial, hemostatic, and astringent effects, used in the treatment of superficial wound bleeding. Silver nitrate application causes eschar, which typically vanishes within several days but may cause permanent skin pigmentation [30,54].

Fibrin Sealant

Fibrin sealants or fibrin glues are adhesive products containing fibrinogen and thrombin [55]. In these products, thrombin converts fibrinogen to fibrin and activates factor XIII to cross-link the fibrin and stabilize the blood clot. The concentrations of fibrinogen and thrombin determine the mechanical strength of the fibrin sealant and the velocity of clot formation, respectively. As fibrin sealants act independently of the coagulation pathways, they may be effective in cases of coagulopathic bleeding. Many clinical trials have demonstrated the safety and efficacy of fibrin sealants in hemorrhage control.

Different fibrin sealant products have been developed with varying thrombin and fibrinogen concentrations, sources of thrombin (bovine, human, and human recombinant), and physical carriers. These differences lead to variable effectiveness of these products in clinical trials and in vivo studies. Fibrin sealants can be divided into solid fibrin sealants and liquid fibrin sealants. TachoSil, Tacho-Comb, fibrin pad, dry fibrin sealant dressing, and Fibriseal are examples of solid fibrin sealant products. Liquid fibrin sealants are provided in two separate vials with a dual-syringe delivery system, in which fibrinogen and thrombin admix immediately before application. Tisseel, Crosseal, Evicel, and Quixil are examples of liquid fibrin sealant products.

Solid fibrin sealants are biodegradable, can be applied with manual pressure, allow cavity packing, can be stored at room temperature, have a long shelf life, and require no preparation before application. However, they are more expensive, and it is difficult to seal a wound with an irregular surface [17,18,31,33,44,56].

Topical Thrombin

Thrombin, formed from prothrombin through the activation of both intrinsic and extrinsic coagulation pathways, converts soluble fibrinogen into insoluble fibrin, forming a fibrin clot. Thrombin has been used as an effective tool to control hemorrhage. Commercial sources of thrombin include bovine-derived thrombin (Thrombin-JMI), human plasma-derived thrombin (Evithrom), and recombinant human thrombin (Recothrom). Randomized, double-blinded clinical trials have found that recombinant human thrombin offers similar safety, comparable efficacy, and less immunologic response than bovine thrombin [17,41]. Hemoblast™ is branded as a three-component hemostatic powder comprising human thrombin, collagen, and chondroitin sulfate [44,57].

Collagen and Microfibrillar Collagen

Collagen, a protein found in humans, activates the hemostatic process [36]. Hait developed microfibrillar collagen (MFC) in 1970. Derived from purified bovine collagen, MFC is a white, dry material used in various forms, powder, sheet, or sponge, to control bleeding. Platelets become entrapped and activated on MFC's surface, leading to coagulation. Due to its reliance on platelet activation, MFC's efficacy is reduced in patients with thrombocytopenia. MFC has been shown to be more effective than oxidized regenerated cellulose (ORC) in reducing bleeding and is marketed as Avitene™ [17,30,50].

Gelatins

Introduced in 1945 by Correll and Wise as hemostatic agents, gelatins are produced from collagen via a hydrolysis reaction and are available as sponges, films, and powders. Mixtures of these powders with sterile saline can be applied as a paste to bleeding sites, absorbing fluid, expanding up to 45 times their dry weight, and forming a physical matrix that acts as a mechanical barrier to bleeding. The neutral pH of gelatins allows them to be effectively used alone or in combination with topical thrombin or other pH-neutral hemostatic agents. Unfortunately, their neutral pH means gelatins have no antimicrobial properties. Marketed gelatin products include Gelfilm and Gelfoam by Pharmacia and Upjohn Company, respectively, as well as FloSeal and Surgiflo, which contain gelatin combined with thrombin [17,30,50,58,59].

Albumin

Albumin, a component of blood, is utilized in various hemostatic materials. Working independently of the coagulation cascade, albumin forms a mechanical sealant at the hemorrhage site. BioGlue, containing bovine albumin (45% solution) and glutaraldehyde (10% solution) in separate reservoirs of a dual cartridge that mixes immediately before application, received FDA approval in 1999. It is an effective hemostatic agent for mechanically reinforcing suture lines in cardiac surgery [17,41,60].

Polyethylene Glycols

Polyethylene glycols are widely used as polymers in the formation of hydrogels. These agents absorb fluid and expand to form a crosslinked hydrogel matrix that creates a mechanical barrier to bleeding. CoSeal and Duraseal, two well-known products based on polyethylene glycol polymers, were approved by the FDA in 2001. Consisting of two solutions of high-molecular-weight polyethylene glycol in sodium phosphate buffer, these solutions are sprayed onto tissues, forming a crosslinked hydrogel matrix. CoSeal does not cause inflammation or produce heat when formed; however, it can swell in the first few days after application, potentially damaging nearby tissue [17,33,35,37,44].

Cyanoacrylates

Alkyl α-cyanoacrylates are liquid biodegradable topical tissue adhesives that polymerize in the presence of water or blood, and hold wound edges together, with bacteriostatic and mechanical hemostatic effects. The alkyl side chain can produce toxic effects, but by increasing the chain length, this toxicity decreases.

Different derivatives of cyanoacrylates have been introduced for hemorrhage management and wound repair. In 1942, ethyl 2-cyanoacrylate was introduced as a glue, and marketed as Superglue and Krazy Glue products. Ethyl cyanoacrylates, if applied on the skin, can form cyanoacetate and formaldehyde, which cause inflammation. In 1998, octyl-2-cyanoacrylate was developed and received FDA approval for wound repair (Dermabond, Ethicon). Butyl-2-cyanoacrylate, another derivative, has been approved for use in Europe and Asia (Histoacryl and Glubran products) [17,36,61-64].

Hydrophilic Polymers With Potassium Salts

Hydrophilic polymers with potassium salts (HPPS) have a hemostatic effect. When the hydrophilic polymers encounter blood, they absorb fluid and concentrate blood components. The aggregation of blood components forms a mechanical barrier to bleeding. The potassium ferrate in these products acts as a binding agent. HPPS products were first marketed as Urgent Quick Release (QR) powder® (Biolife, Sarasota, FL), Bioseal® (Biolife, Sarasota, FL), and Wound Seal, Pro QR Powder®. As HPPS are not absorbable and work independently of the body’s innate coagulation system, they can be used in patients who consume anticoagulant and antiplatelet drugs [30].

Bone Wax

A mixture of beeswax, salicylic acid, and almond oil was first used in 1866 as a hemostatic agent for skull bleedings of animals. At present, the composition of this nonabsorbable mixture has changed to beeswax, paraffin, isopropyl palmitate, and a wax-softening agent. This mixture, branded as “bone wax” and marketed by Johnson and Johnson, Ethicon Inc., acts mechanically through the occlusion of bleeding channels on the cut bone channel. As bone wax hinders osteogenesis and thus impairs bone healing, its use is not recommended in most cases [17,65].

Ostene

Ostene is a biocompatible and absorbable hemostatic agent that was first introduced in 2001 as a bone wax alternative. Its hemostatic mechanism of action is similar to bone wax. Ostene is a water-soluble alkylene oxide block copolymer (poloxamers or Pluronics) approved by the FDA for control of bleeding from bone surfaces. Ostene is preferred to bone wax because it does not impair bone healing and is eventually absorbed. Ostene is currently marketed by Ceremed Inc. [17,65,66].

Future perspectives

Recent studies focus on developing new hemostatic agents based on nanotechnology, as well as combining hemostatic agents to enhance their efficiency and introduce new features. Examples include liposome-based nanoparticles (LNPs), graphene-montmorillonite composite sponges, and silica or chitosan nanoparticles loaded with caffeic acid [67,68].

## Conclusions

In traumatic hemorrhage, prognosis depends on the time to medical treatment and prehospital control of the bleeding. Effective and high-performance hemostats are needed to control bleeding and reduce traumatic death. An ideal hemostatic agent would be one that is easy to use, highly effective, nonantigenic, fully absorbable, safe, and reasonably priced. Currently, various hemostatic agents containing thrombin, chitosan, collagen, fibrin, and silicate-based materials, among others, are available. The mechanism of chitosan involves adhering to tissues and sealing vessels; however, its effectiveness diminishes over time. Silicate-based mineral powders function by rapidly absorbing water, concentrating clotting factors and cells, and promoting clot formation. Concentrated fibrinogen and thrombin are used to create a dry fibrin sealant dressing, but their cost and more stringent testing standards prevent widespread use. In some body areas, narrow but deeply penetrating wounds respond well to injectable expandable sponges. Caustic agents are effective in coagulating blood proteins, but they lead to tissue necrosis and form eschar.

These products have many different limitations, such as risk of infection, immunogenicity, tissue damage, limited usage and efficacy, high costs, short shelf life, and the requirement for storage under specific conditions. Therefore, future studies should be considered to overcome these limitations and develop multifunctional effective hemostatic materials for widespread usage.
